# Rapid and sensitive detection of canine distemper virus by one-tube reverse transcription-insulated isothermal polymerase chain reaction

**DOI:** 10.1186/s12917-014-0213-8

**Published:** 2014-09-09

**Authors:** Rebecca P Wilkes, Yun-Long Tsai, Pei-Yu Lee, Fu-Chun Lee, Hsiao-Fen Grace Chang, Hwa-Tang Thomas Wang

**Affiliations:** University of Tennessee College of Veterinary Medicine Clinical Virology Lab, Department of Biomedical and Diagnostic Sciences, University of Tennessee, 2407 River Dr, Rm A205, Knoxville, TN 37996 USA; GeneReach USA, Lexington, MA USA

**Keywords:** Canine distemper virus, Insulated isothermal polymerase chain reaction, iiPCR, Point-of-care diagnosis

## Abstract

**Background:**

Canine distemper virus (CDV) has been associated with outbreaks of canine infectious respiratory disease in shelters and boarding kennel environments. POCKIT^TM^ Nucleic Acid Analyzer is a field-deployable device capable of generating automatically interpreted insulated isothermal polymerase chain reaction (iiPCR) results from extracted nucleic acid within one hour. In this study, reverse transcription iiPCR (RT-iiPCR) was developed to facilitate point-of-need diagnosis of CDV infection.

**Results:**

Analytical sensitivity (limit of detection 95%) of the established CDV RT-iiPCR was about 11 copies of *in vitro* transcribed RNA per reaction. CDV RT-iiPCR generated positive signals from CDV, but not *Bordetella bronchiseptica*, canine parvovirus, canine herpesvirus, canine adenovirus 2, canine influenza virus (subtype H3N8), canine parainfluenza virus, and canine respiratory coronavirus. To evaluate accuracy of the established reaction in canine distemper clinical diagnosis, 110 specimens from dogs, raccoons, and foxes suspected with CDV infection were tested simultaneously by CDV RT-iiPCR and real-time RT-PCR. CDV RT-iiPCR demonstrated excellent sensitivity (100%) and specificity (100%), compared to real-time RT-PCR.

**Conclusions:**

The results indicated an excellent correlation between RT-iiPCR and a reference real time RT-PCR method. Working in a lyophilized format, the established method has great potential to be used for point-of-care diagnosis of canine distemper in animals, especially in resource-limited facilities.

## Background

Canine distemper (CD), caused by canine distemper virus (CDV), is a highly contagious and lethal systemic disease in young dogs [1]. This virus infects and produces clinical disease in members of the order *Carnivora*, including members of the families *Canidae* (fox, coyote, wolf), *Mustelidae* (ferret, skunk, badger, mink, weasel, otter, fisher), *Procyonidae* (raccoon), *Felidae* (lions, tigers), *Ailuridae* (red panda), *Hyaenidae* (hyenas), *Ursidae* (bear), and *Viverridae* (palm civet, genets) [2].

CDV is a viral disease of global importance in common and endangered carnivores [3]. CDV contributed to the endangerment of the North American black-footed ferret [4], is an emerging disease in the endangered wild Amur tigers in the Russian Far East [3], and led to the death of 20% of the Serengeti and Mara lions in the 1990s [5]. The virus also causes disease in non-carnivores, including collared peccaries (*Tayassu tajacu*) [6], and has recently been detected in macaques (*Macaca*) in Japan [7] and China [8].

Raccoons have been implicated in the spread of CDV to animals in zoo collections and conservation parks [2], and CDV outbreaks have been documented in raccoons living around a suburban zoo [9]. Therefore, domestic dogs are not the only species at risk following the movement of the distemper virus into a wildlife species. CDV produced mass die-offs of thousands of Caspian seals (*Pusa caspica*) between 1997 and 2001. The source for the epizootic strain is still unknown but thought to be a terrestrial carnivore [10].

CDV, a member of *Morbillivirus* genus in the *Paramyxoviridae* family, contains a single-stranded, negative-sense genomic RNA, which is around 15,690-nt long [11,12]. CDV infection leads to a multi-systemic infection with signs including respiratory, gastrointestinal and neurological disorders. CDV has been classified into several genetic lineages based on diversity among strains from distinct geographic locations. There are 7 major CDV lineages, America-1, America-2, European, European Wildlife, Asia-1, Asia-2, and Arctic like [13], as well as some additional proposed ones that have been more recently discovered, including Asia-3 [14], Asia-4 [15], Africa [13], Europe/South America 1 (European), South America 2, and South America 3 [16-19]. The Onderstepoort and Snyder Hill strains, members of the America-1 lineage, were used widely to produce the distemper vaccines in the 1950s, and these are the same vaccines used today.

Several recent reports suggest both the re-emergence and increased activity of CDV worldwide [20]. Cases of CD have also been reported this decade in vaccinated dogs in Japan [21], Mexico [22], Argentina [16], and the United States [23]. In each case, the strains associated with these cases have been shown to be genetically divergent from the vaccine lines. Respiratory and enteric signs associated with CD may be confused with those of other respiratory and enteric diseases, hindering early CDV diagnosis [24,25]. Therefore, rapid and accurate diagnosis of CDV infection would enable veterinarians to implement appropriate strategies in time to improve disease management and prevent outbreaks, particularly within a shelter environment.

Virus isolation has previously been difficult due to the lack of an efficient cell line. Engineered VERO cells have been developed to express the canine signaling lymphocyte activation molecule (SLAM; also known as CD150), which is one of the cellular receptors for canine distemper virus (CDV). These engineered cells show cytopathic effects around one day after inoculation and are highly sensitive for CDV isolation from canine hosts [26]. However, there is species-preference for the type of SLAM receptor [27], which has the potential to affect the ability to isolate the virus from certain non-carnivore hosts. Additionally, this method requires a cell culture system and is relatively expensive and labor intensive. CDV immunoassays for detection of CDV antigen, such as immunofluorescence staining and ELISA [28-32], in general are not sensitive enough to detect CDV in asymptomatic dogs or during acute CD infection [31]. Various conventional, nested, and real-time reverse transcription polymerase chain reaction (RT-PCR) assays, which offer high degrees of detection sensitivity and specificity, have also been developed for CDV [33-42]. However, requiring an experienced technician and a relatively expensive thermocycler, these assays have been applied to CDV diagnosis mostly in professional laboratories. As a result, a rapid, affordable, and user-friendly platform is still needed for early point-of-care (POC) detection of CDV infection.

**Figure 1:**
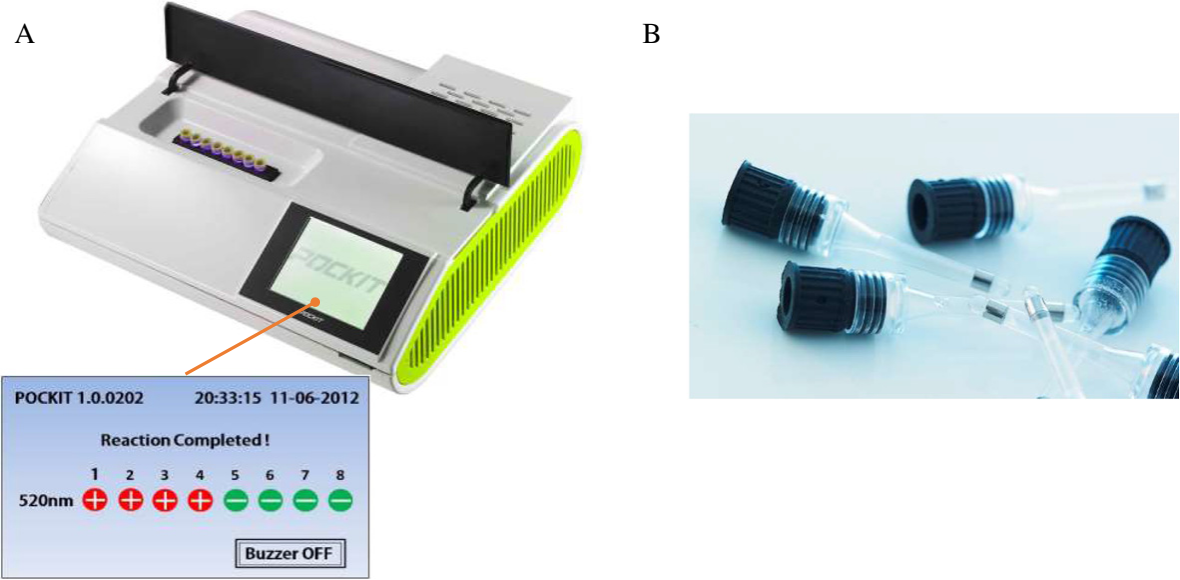
**Photograph of POCKIT™ device and R-tube™.** The POCKIT™ device **(A)** is small (28 × 25 × 8.5 cm, W × D × H) and simple to operate. With a single default program, amplification and product detection steps of both iiPCR and RT-iiPCR could be completed on this device within 1 hour. One to eight reactions could be performed simultaneously in one run. Its simple user interface is accessible via a color touch screen. The reaction is carried out in a R-tube™ **(B)**, a capillary tube wrapped with a copper ring near its bottom end.

Recently, insulated isothermal PCR (iiPCR), based on Rayleigh-Bénard convection PCR [43], has been shown to achieve relatively consistent and fast target amplification by convective PCR. The reaction is done in a copper ring-wrapped capillary tube heated within a thermally baffled device, which is relatively simple and inexpensive [44,45]. In Rayleigh-Bénard convection PCR [43], spontaneous fluid convection in a cylindrical vessel is driven by temperature gradients that are formed simply by heating the vessel from the bottom at a fixed temperature. Reaction constituents are circulated sequentially through zones of temperature gradients to allow the denaturation, annealing, and extension steps of PCR to take place. Because each cycle is estimated to complete within a few seconds, detectable amounts of amplicons could be produced within 30 minutes. For iiPCR, the reaction is performed in a special capillary tube (namely R-tube™, Figure 1), which is wrapped with a copper ring near its base. This design facilitates fast and consistent heating of the fluid at the bottom end of capillary tubes. Furthermore, fluorescent hydrolysis probe technology was integrated into iiPCR to eliminate post-amplification analysis of amplicons [46]. POCKIT™ Nucleic Acid Analyzer (POCKIT™; GeneReach, Taichung City, Taiwan) is now available commercially to perform iiPCR/reverse transcription (RT)-iiPCR amplification, signal detection and data interpretation (Figure 1). Its temperature program allows single-tube RT-iiPCR. Fluorescent signals generated from fluorescent probe hydrolysis are detected and converted to digital signals by the optical and data processing modules integrated in the device. The built-in algorithms determine the signal-to-noise (S/N) ratio, *i.e.* fluorescence_after iiPCR_/fluorescence_before iiPCR_ [46] of the reaction and convert them automatically into simple readouts (“+”, “−“, or “?”) based on the default thresholds. The results are shown on the display screen within one hour. Validation of an iiPCR assay for white spot syndrome virus (WSSV), a shrimp DNA virus, demonstrated that this system could provide results with sensitivity and specificity comparable to those of a nested PCR assay within 1 hour for the detection of WSSV in shrimp samples [47].

Taking advantage of the hydrolysis probe-iiPCR method and the POCKIT™ device, the aim of this study was to develop a rapid, sensitive and specific method for CDV detection for points of care. The established CDV RT-iiPCR method was compared to a real-time RT-PCR [42] to evaluated its limit of detection as well as sensitivity and specificity for the detection of CDV in clinical specimens using samples collected from dogs, raccoons, and foxes suspected to have CDV infection.

## Results

### CDV RT-iiPCR sensitivity and specificity

The percentage of positive results from *in vitro* transcribed RNA dilutions were 100% (21/21), 100.0% (21/21), 95% (20/21), 25.0% (4/16), and 0% (0/16) for 10^3^, 10^2^, 10^1^, 10^0^, and 10^-1^ copies of the RNA standard, respectively. The ≥95% detection rate and 95% confidence limits, calculated by probit regression analysis, were 10.1 and 7.6–15.6 copies per reaction, respectively, indicating that the accuracy of the established reaction was comparable to that of the real-time RT-PCR for the detection of CDV.

Dilutions of the Onderstepoort reference strain, when tested with both real-time RT-PCR and RT-iiPCR, generated positive results with all 10^-2^ dilution samples. At the 10^-3^ dilution, 2/3 and 1/2 samples showed positive signals in RT-iiPCR and real-time RT-PCR, respectively (Table 1), indicating that the detection limit of RT-iiPCR was comparable to that of real-time RT-PCR.

**Table 1:** **Comparison of analytical sensitivity between validated CDV real-time RT-PCR and RT-iiPCR using viral RNA of Onderstepoort strain**

**Sample dilution**	**Real-time RT-PCR**		**RT-iiPCR No. positive/total**
	**No. positive/total**	**Ct**	
NTC*	-		-
Positive control	+		+
10^-1^	2/2	34.09	3/3
10^-2^	2/2	36.99	3/3
10^-3^	1/2	40.2	2/3
10^-4^	0/2		0/3
10^-5^	0/2		0/3
10^-6^	0/2		0/3

*NTC-No template control.

During specificity testing, positive signals were generated only from CDV (Snyder Hill strain) but not from the other canine pathogens tested (Table 2), indicating that the established reaction could detect CDV specifically.

**Table 2:** **Exclusivity analysis of CDV RT-iiPCR**

**Pathogen**	**Source**	**RT-iiPCR**		**Real-time RT PCR (Ct)**
		**Results**	**S/N**	
Canine distemper virus	Snyder Hill strain (ATCC)	+	1.94	34.45
*Bordetella bronchiseptica*	Clinical sample*	-	0.93	Neg
Canine herpesvirus	ATCC strain	-	0.97	Neg
Canine respiratory coronavirus	Clinical sample*	-	0.90	Neg
Canine parainfluenza virus	ATCC strain	-	0.93	Neg
Canine adenovirus 2	ATCC strain	-	0.93	Neg
Canine parvovirus	Cornell strain	-	0.94	Neg
Influenza virus H3N8	Clinical sample*	-	0.93	Neg

*Pathogens obtained from clinical samples were previously cultured/isolated and validated by sequencing by the UTCVM Clinical Bacteriology and Virology Labs.

### Validation of CDV RT-iiPCR using clinical samples

A clinical evaluation was further performed to determine whether the CDV RT-iiPCR was suitable for detection of CDV viruses from clinical samples. For this purpose, 110 clinical samples submitted to the University of Tennessee, College of Veterinary Medicine (UTCVM), Clinical Virology Lab from 2010 to 2014 were tested by RT-iiPCR and real-time RT-PCR. These samples included strains from 4 different clades of the phylogenetic trees derived from sequences of the *H* gene and *M-F* intergenic region [48]. 100% agreement was found between the two reactions (56 positive and 54 negative) (Table 3). No discrepancy was found in samples (13/56) containing low levels of CDV (Ct > 35, real-time RT-PCR, indicating that the established method reliably detects low amounts of CDV in clinical samples. Positive samples had real-time RT-PCR Ct values ranging from 14.31 to 39.41, indicating that the RT-iiPCR was able to detect viral RNA across the entire range of the assay.

**Table 3:** **Comparison of CDV RT-iiPCR versus CDV real-time RT-PCR for the detection of CDV in 110 samples**

		**Real-time RT-PCR**		
		**Positive**	**Negative**	**Total**
RT-iiPCR	Positive	56	0	56
	Negative	0	54	54
	Total	56	54	110

## Discussion

In this report, aimed to improve point-of-care diagnosis of CD, a novel one-tube RT-iiPCR method performed in a relatively simple field-deployable device, POCKIT™ Nucleic Acid Analyzer, was established for the detection of CDV in clinical samples. The results show that the RT-iiPCR was a sensitive and specific test to confirm CDV infection in dogs with detection limits comparable to that of the reference real-time RT-PCR.

Molecular methods such as nested or real-time PCR assays require experienced technicians and costly equipment, limiting their application to professional laboratories. Moreover, the nested PCR assay is quite laborious and time-consuming and requires gel electrophoresis for amplicon detection, making it prone to cross contamination. Recently developed miniaturized chips have progressed by the introduction of microfluid technology for pathogen detection [49-51]. However, high costs of the reaction vessels and instruments, problems in product manufacturing, and sub-optimal detection methods have deferred these methods from being applied to point-of-care diagnostics for resource-poor facilities.

In convective PCR, PCR cycling could be accomplished at high speeds using a single simple heater. Combining iiPCR and 5′ nuclease-based signal detection, automatically interpreted results of the established CDV reaction were generated within one hour in the POCKIT™ device. Hence, taking sample collection and nucleic acid extraction into account, the procedure could be completed within 1.5 hours. Both cDNA synthesis and PCR amplification are performed in a single step, eliminating the additional pipetting steps as well as minimizing the risks of contamination. Furthermore, different types of clinical samples (including swabs, urine, and tissues) were shown to be suitable sample types for CDV RT-iiPCR in this study. Purification of nucleic acids before PCR in general is required for successful target amplification because Taq DNA polymerase used in PCR is sensitive to inhibitors originating from samples and/or the purification processes [52]. Inhibitors often decrease assay sensitivity or even lead to false-negative results. For easy preparation of nucleic acids for CDV RT-iiPCR at POC settings, PetNAD™ Nucleic Acid Co-prep kit (GeneReach USA), a column-based simple nucleic acid extraction method, is an option for whole blood, urine, oropharyngeal swab, conjunctival swab, and nasal swab samples.

The POCKIT™ system is a field portable device, which is enabled by its small size (28 × 25 × 8.5 cm, W × D × H), light weight (2.1 kg), and ruggedness. The device has passed the atmospheric preconditioning, atmospheric conditioning, compression, vibration, shock, and vibration tests specified in the International Safe Transit Association (ISTA) Procedure 2A 2011. Another important feature for the POCKIT™ device to be field deployable is that it could be operated with a rechargeable battery or car battery, allowing application of this system in low resource settings with unstable electricity supply. Furthermore, working in a lyophilized format, the reagents could be shipped and stored without the need for refrigeration.

CDV is not a zoonotic pathogen and pathological waste in general could be placed in a plastic bag to be disposed of as regular solid waste (www.epa.state.oh.us/dsiwm). Furthermore, disposables (such as R-tube™, Eppendorf tubes, and micropipette tips) used in the RT-iiPCR/POCKIT™ tests are of small sizes. Therefore, for tests performed outside of the office/laboratory, infectious wastes generated from this test could be packed with double leak-proof plastic bags or biohazard bags to be treated locally or taken back to the office/laboratory for disposal. The disposal should follow the current Biosafety guidelines.

Instead of sending specimens to a diagnostic laboratory, on-site diagnosis of CDV infection could eliminate shipping and processing costs and shorten test turn-around time significantly to improve disease management. For canine infectious respiratory disease in shelters and boarding kennel environments, this would allow appropriate quarantine and management of infected dogs. Additionally, this method identifies a conserved region of the genome and was used to successfully detect CDV in tissues from raccoons and foxes in this study. Therefore, the established method could be utilized potentially in the field for CDV surveillance in wildlife populations.

## Conclusions

In summary, the novel features of the CDV RT-iiPCR with POCKIT™ method enabled automatic result interpretation of CDV RNA detection in a portable device with sensitivity and specificity comparable to real-time RT-PCR. This field-deployable assay could serve as a useful tool for point-of-care needs in CDV diagnosis and surveillance.

## Methods

### Samples

Reference strains used included the Snyder Hill strain (ATCC, Manassas, VA, USA), an isolate from the brain of a dog in Ithaca, New York, in 1950s and the Onderstepoort strain, originating from a disease outbreak among North American ranched foxes in the 1930s.

Clinical samples used were samples submitted to the UTCVM Clinical Virology Lab between 2010 and 2014 for real-time RT-PCR analysis for CDV. Submissions were mainly from eastern Tennessee, but samples from Canada, Texas, Washington, Kentucky, West Virginia, Virginia, South Carolina, and Pennsylvania were also tested. Most samples were from dogs (including nasal/oropharyngeal swabs and urine), but tissues from raccoons and foxes were also included. These samples were diagnostic samples submitted to the lab for routine testing and not collected specifically from research animals for research purposes; therefore, this was exempt from requiring Institutional Animal Care and Use Committee approval.

### RNA extraction

RNA was extracted from clinical samples (including nasopharyngeal/conjunctival swabs, urine, and tissues) and dilutions of reference strains with the QIAamp viral RNA kit (Qiagen, Valencia, CA, USA) according to the manufacturer’s instructions. Tissues were macerated with an equal volume of PBS in a Stomacher (Tekmar Company, Cincinnati, OH, USA) and supernatant was used for extraction. Samples previously extracted were stored at -80°C.

### Plasmid construction and standard RNA preparation

To generate the standard template (pT-CDV), a 1463-bp partial cDNA sequence of the nucleocapsid (*N*) gene amplified by RT-PCR (primers CDV F1 and CDV R1463, Table 4) was cloned into the pCR2.1TOPO vector (Invitrogen, Carlsbad, CA). Calculation of copy number was based on the concentrations determined by a DU730 spectrophotometer (Beckman Coulter, Brea, CA, USA). Dilutions of standard DNA were prepared in TE buffer (10 mM Tris–HCl and 1 mM EDTA, pH 8.0) containing 30 ng/μl tRNA from *Escherichia coli* (Sigma-Aldrich, Milan, Italy) and stored at -20°C. To prepare the RNA standards, *Hin*dIII-linearized pT-CDV plasmid was subjected to *in vitro* transcription using the MEGAscript T7 Kit (Ambion, Huntingdon, UK). RNA transcripts were produced, treated with DNase I, and purified by LiCl precipitation by following the manufacturer’s instruction. Copy numbers of RNA transcripts were calculated based on concentrations determined by a spectrophotometer. Dilutions of RNA were prepared in TE buffer containing 30 ng/μl tRNA. Aliquots were frozen at −70°C and used only once.

**Table 4:** **Primers and probes used in cloning, CDV RT-iiPCR and real-time RT-PCR**

**Primer**	**Nucleotide sequence (5′–3′)**	Note*
CDV-F	AGCTAGTTTCATCTTAACTATCAAATT	CDV real-time RT-PCR forward primer^a^
CDV-R	TTAACTCTCCAGAAAACTCATGC	CDV real-time RT-PCR reverse primer^a^
CDV-P	FAM-ACCCAAGAGCCGGATACATAGTTTCAATGC-BHQ1	CDV real-time RT-PCR probe^a^
CDV-F1	ATGGCTAGCCTTCTTAAAAGCCTCACA	CDV cloning primer (nt108-134)*
CDV-R1	TTCCGATCATCGTCATTTCCATCA	CDV cloning primer (nt1570-1547)*
CDViiF	CCGGAAATCAACGGACCTAAATT	CDV RT-iiPCR primer (nt291 ~ 313)*
CDViiR	GTCGTCTATGATCCTCTGGATCAA	CDV RT-iiPCR primer (nt389 ~ 366)*
CDViiP	FAM-CAGTATCCTCTCCTTGTTCGTGGA-MGB-NFQ	CDV RT-iiPCR probe (nt329 ~ 352)*

*Nucleotide position is based on GenBank accession no. AF305419.1; ^a^Elia *et al*. [42].

### CDV real-time RT-PCR

RNA samples were tested in the UTCVM Clinical Virology Lab by real-time RT-PCR using the primers and probe designed by Elia *et al*. [42] (Table 4). Briefly, the SuperScript III Platinum One-Step qRT-PCR Kit (Invitrogen, Life Technologies, Grand Island, NY, USA) was used with 300 nM of each primer and 200 nM of probe in a 25 μL total volume reaction with 5 μL RNA. Amplification was carried out in a Smart Cycler II (Cepheid, Sunnyvale, CA, USA) with an initial RT step of 42°C for 30 min., followed by 95°C for 2 min., and 45 cycles of 95°C for 15 sec., 48°C for 1 min. and 60°C for 1 min.

### CDV RT-iiPCR

The CDV RT-iiPCR was designed on the basis of the hydrolysis probe-based POCKIT™ method described previously [46]. The CDV-specific RT-iiPCR primers (Table 4) were designed on the basis of 90 CDV N sequences available in GenBank. These sequences included strains collected from dogs, wolves (GenBank accession no. KF914669.1), foxes (GenBank accession no. JX681125, EU489475.1, EF445056.1, EF445050.1, FJ710174.1, and HQ540293.1), and mink (GenBank accession no. EU375802.1). The *N* gene contains conserved sequences with the highest homology within the genomic RNA of CDV. Primer and probe pairs were designed according to the recommended principles for iiPCR (http://www.iipcr.com/eweb/uploadfile/20130522114104277.pdf). Amplicons with major secondary structures were excluded, based on prediction made by the MFold program (http://mfold.rna.albany.edu/?q=mfold). The probe was labeled with fluorescent reporter dye 6-carboxyfluorescein (FAM) at the 5′ end and a minor groove binder group (MGB) with a non-fluorescent quencher (NFQ) at the 3′ end (Applied Biosystems) (Table 4). The MGB group increased the stability and specificity of probe hybridization [53]. Annealing temperature of the probe was approximately 10°C higher than those of the iiPCR primers. The expected size of the amplicon was 99 bp. Components of the iiPCR reaction were optimized and the final master mix included the following: forward primer CDViiF, reverse primer CDViiR, probe CDViiP, dNTP, Taq DNA polymerase, MMLV reverse transcriptase, 50 mM Tris–HCl (pH 8.3), 75 mM KCl, 3 mM MgCl_2_, and 1 mM DTT. These components were lyophilized. The lyophilized reagent was rehydrated with 50 μL of Reconstitution Buffer (GeneReach USA) before 5 μL of the nucleic acid sample was added to the mixture. Subsequently, 50 μL of the final mixture was transferred to an R-tube™ (GeneReach USA), which was spun briefly in a cubee™ mini centrifuge (GeneReach USA) and placed into the reaction chamber of POCKIT™ Nucleic Acid Analyzer. The reaction completed in less than one hour and showed the results on the display screen. Using an optical detection module similar to a previously described iiPCR device [46], POCKIT™ collects optical signals through an integrated circuits controller-regulated sensor. The collected fluorescent signal-to-noise ratios (signal_after_/signal_before_) were converted to positive (+symbol), negative (- symbol), and indeterminate (? symbol) according to the default S/N thresholds [44,46]. A “?” result indicated that the signals were ambiguous and the sample should be tested again.

### CDV RT-iiPCR sensitivity and specificity

Analytical sensitivity of the final CDV RT-iiPCR was determined by using 10-fold serial dilutions of *in vitro* transcribed RNA. Each dilution of the *in vitro* transcribed RNA was tested 16 or 21 times. To further evaluate the sensitivity of the CDV RT-iiPCR, triplicates of 10-fold serial dilutions of CDV Onderstepoort strain were subjected to RNA extraction and analyzed by RT-iiPCR and the reference real-time RT-PCR in parallel.

To evaluate specificity of CDV RT-iiPCR, nucleic acid extracts of common canine respiratory pathogens, including *Bordetella bronchiseptica*, canine parvovirus, canine herpesvirus, canine adenovirus 2, canine influenza virus (subtype H3N8), canine parainfluenza virus, and canine respiratory coronavirus, were subjected to CDV RT-iiPCR analysis. RNA extracted from the Snyder Hill reference strain was used as a positive control in this experiment.

## Abbreviations

CD: Canine distemper
CDV: Canine distemper virus
FAM: 6-carboxyfluorescein
iiPCR: Insulated isothermal PCR
MGB: Minor groove binder group
NFQ: Non-fluorescent quencher
RT-iiPCR: Revers transcription-insulated isothermal PCR
S/N: Signal-to-noise ratio
UTCVM: University of Tennessee College of Veterinary Medicine
